# Supplementary material for: Epigenetic Responses to Abusive versus Accidental Injuries in Children: A Cross-sectional Epigenome Wide Association Meta-analysis
Source: medRxiv. 2026 Mar 1:2026.02.02.26345419. Originally published 2026 Feb 4. Preprint. [Version 2] doi: 10.64898/2026.02.02.26345419 (PMC12889775; doi:10.64898/2026.02.02.26345419)
Supplement: 1 [file NIHPP2026.02.02.26345419V2-supplement-1.pdf]

medRxiv preprint doi: https://doi.org/10.64898/2026.02.02.26345419; this version posted March 1, 2026. The copyright holder for this preprint (which was not certified by peer review) is the author/funder, who has granted medRxiv a license to display the preprint in perpetuity.
It is made available under a CC-BY 4.0 International license .

Supplementary Figures and Table

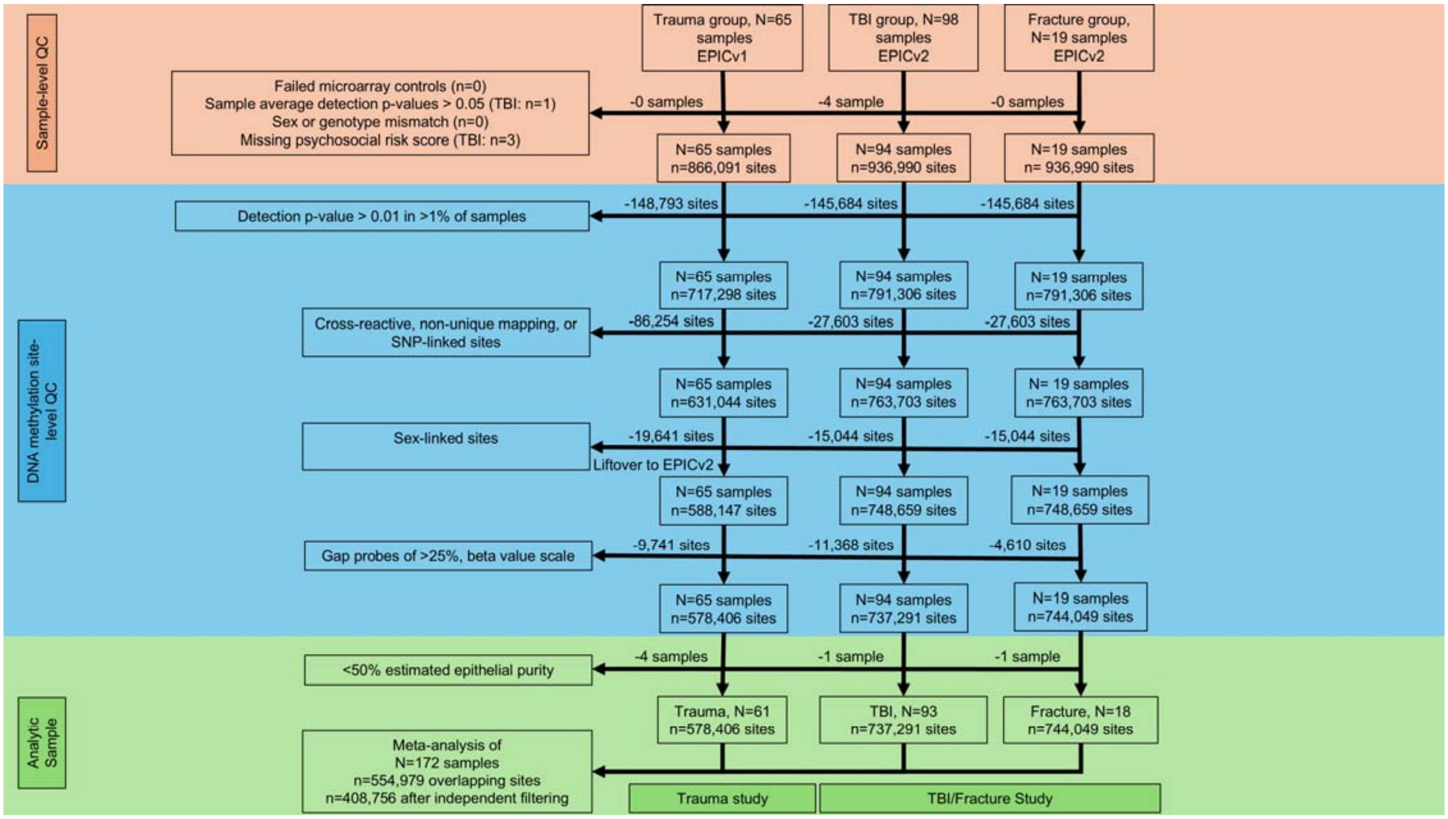

Supplementary Figure 1. Overview of quality control procedures presented as a flow chart for each study sample. Sample-level quality control exclusions painted in orange. DNA methylation site-specific exclusions painted in blue. Final analytic sample after exclusion of low predicted epithelial cell origin painted in blue.
