# Supplementary material for: Epigenetic Responses to Abusive versus Accidental Injuries in Children: A Cross-sectional Epigenome Wide Association Meta-analysis
Source: medRxiv. 2026 Feb 4:2026.02.02.26345419. Preprint. [Version 1] doi: 10.64898/2026.02.02.26345419 (PMC12889775; doi:10.64898/2026.02.02.26345419)
Supplement: Supplementary file 1 [file NIHPP2026.02.02.26345419V1-supplement-1.pdf]

medRxiv preprint doi: https://doi.org/10.64898/2026.02.02.26345419; this version posted February 4, 2026. The copyright holder for this preprint (which was not certified by peer review) is the author/funder, who has granted medRxiv a license to display the preprint in perpetuity.
It is made available under a CC-BY 4.0 International license .

Supplementary Figures and Table

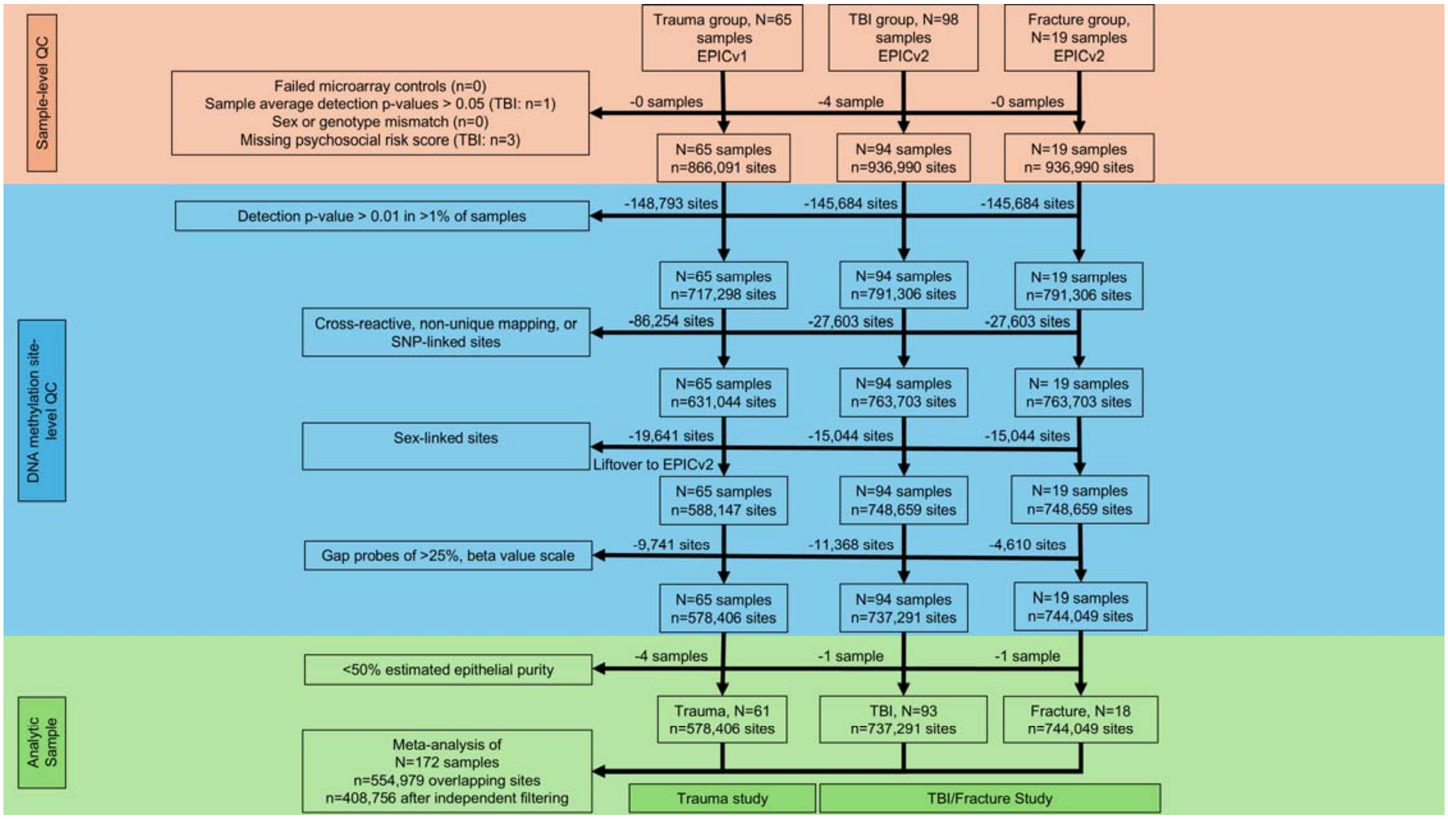

Supplementary Figure 1. Overview of quality control procedures presented as a flow chart for each study sample. Sample-level quality control exclusions painted in orange. DNA methylation site-specific exclusions painted in blue. Final analytic sample after exclusion of low predicted epithelial cell origin painted in blue.

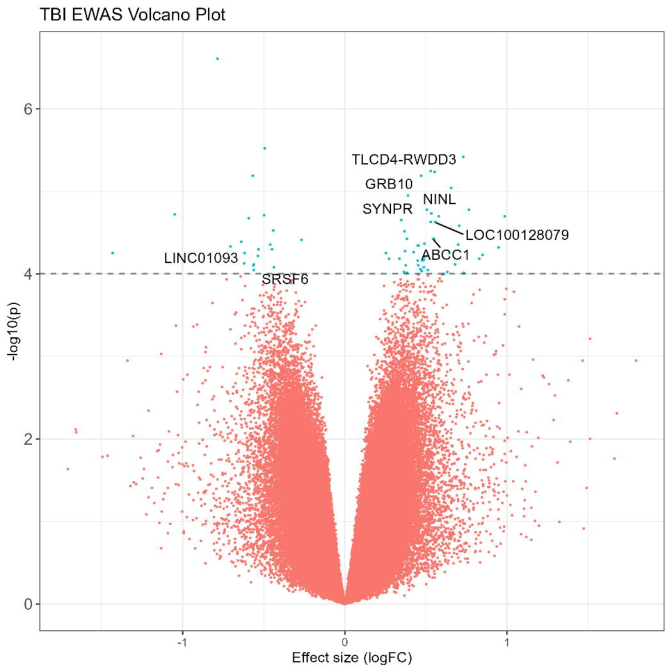
TBI EWAS Volcano Plot
TLCD4-RWDD3
GRB10
NINL
SYNPR
LOC100128079
ABCC1
LINC01093
SRSF6
6
4
2
0
-log10(p)
-1
0
1
Effect size (logFC)

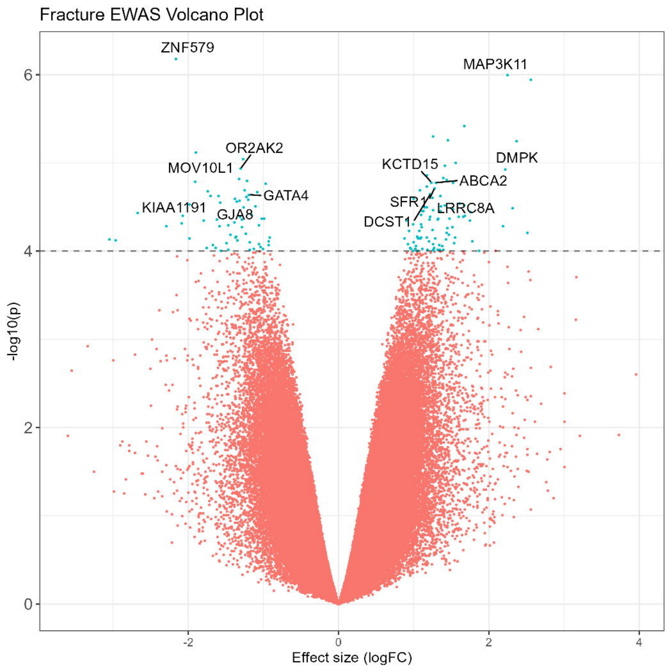

Fracture EWAS Volcano Plot
ZNF579
MAP3K11
OR2AK2
MOV10L1
GATA4
KIAA1191
GJA8
KCTD15
ABCA2
DMPK
SFR1
LRRC8A
DCST1
-log10(p)
Effect size (logFC)
6
4
2
0
-2
0
2
4

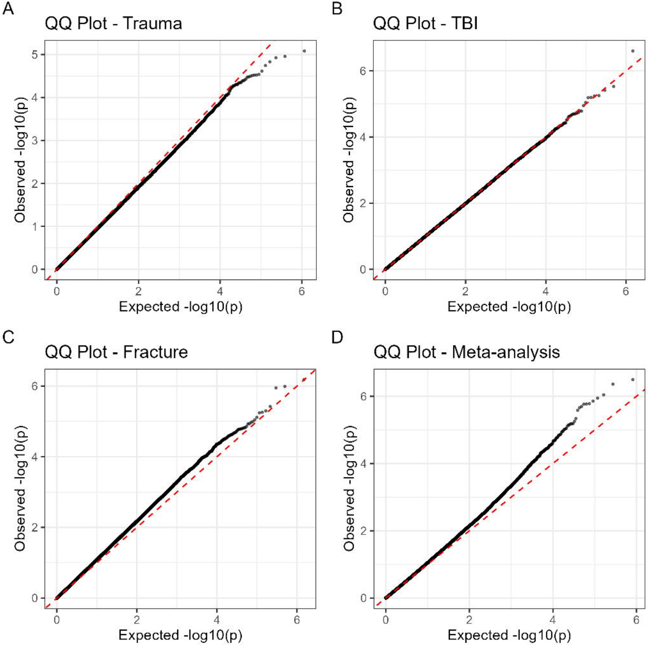
A
QQ Plot - Trauma
Observed -log10(p)
Expected -log10(p)
B
QQ Plot - TBI
Observed -log10(p)
Expected -log10(p)
C
QQ Plot - Fracture
Observed -log10(p)
Expected -log10(p)
D
QQ Plot - Meta-analysis
Observed -log10(p)
Expected -log10(p)

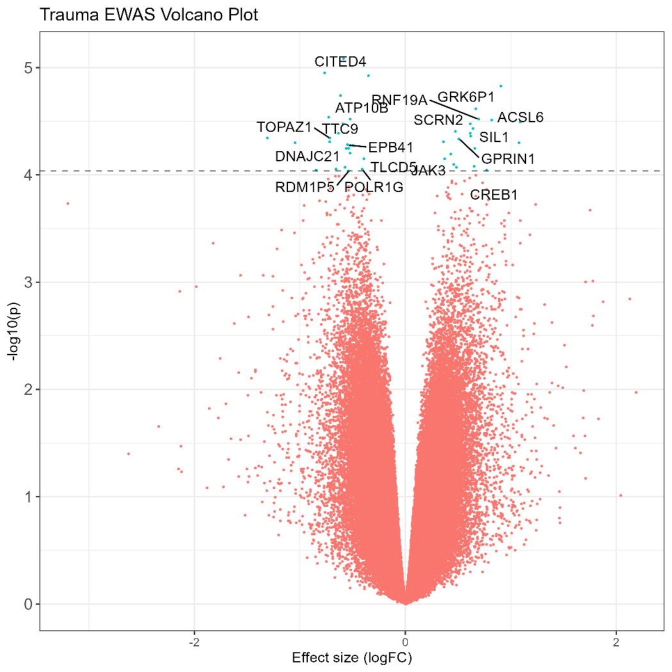

Trauma EWAS Volcano Plot
CITED4
GRK6P1
RNF19A
ATP10B
ACSL6
SCRN2
TOPAZ1
TTC9
SIL1
EPB41
DNAJC21
GPRIN1
TLCD5
JAK3
RDM1P5
POLR1G
CREB1
-log10(p)
Effect size (logFC)
5
4
3
2
1
0
-2
0
2
